# Luminescence Mechanism of Carbon Dots by Tailoring Functional Groups for Sensing Fe^3+^ Ions

**DOI:** 10.3390/nano8040233

**Published:** 2018-04-12

**Authors:** Jingjing Yu, Chang Liu, Kang Yuan, Zunming Lu, Yahui Cheng, Lanlan Li, Xinghua Zhang, Peng Jin, Fanbin Meng, Hui Liu

**Affiliations:** 1 School of Materials Science and Engineering, Hebei University of Technology, Tianjin 300130, China; yujingjing2016@gmail.com (J.Y.); liuchang6212@gmail.com (C.L.); yuankang2016@gmail.com (K.Y.); luzunming@hebut.edu.cn (Z.L.); Liabc@hebut.edu.cn (L.L.); mengfanbing236@gmail.com (F.M.); 2 Department of Electronics and Key Laboratory of Photo-Electronic Thin Film Devices and Technology of Tianjin, Nankai University, Tianjin 300071, China; chengyahui@nankai.edu.cn; 3 Key Laboratory for Advanced Ceramics and Machining Technology of Ministry of Education, the Institute for New Energy Materials, School of Materials Science and Engineering, Tianjin University, Tianjin 300072, China

**Keywords:** carbon dots, microwave method, surface state, luminescence mechanism, ion detection

## Abstract

In this paper, spherical carbon dots (CDs) with distinct compositions and surface states have been successfully synthesized by a facile microwave method. From the fluorescence spectra, several characteristic luminescence features have been observed: surface amino groups are dominant in the whole emission spectra centering at 445 nm, and the fingerprint emissions relevant to the impurity levels formed by some groups related to C and N elements, including C-C/C=C (intrinsic C), C-N (graphitic N), N-containing heterocycles (pyridine N) and C=O groups, are located around 305 nm, 355 nm, 410 nm, and 500 nm, respectively. Those fine luminescence features could be ascribed to the electron transition among various trapping states within the band structure caused by different chemical bonds in carbon cores, or functional groups attached to the CDs’ surfaces. According to the theoretical calculations and experimental results, a scheme of the band structure has been proposed to describe the positions of those trapping states within the band gap. Additionally, it has also been observed that the emission of CDs is sensitive to the concentration of Fe^3+^ ions with a linear relation in the range of Fe^3+^ concentration from 12.5 to 250 μM.

## 1. Introduction

As an emerging new class of nanomaterials, carbon dots have attracted much research interest recently due to their unique optical properties, abundant raw materials, simple preparation methods, basically non-toxic, good biocompatibility, and easy surface modification [1,2,3]. Since their discovery, CDs have been applied in various technical fields, such as bio-imaging [4,5], light emitting diodes [6,7], photo-catalysts [8,9], anticancer drug delivery [10,11], chemical sensors (metal ions [12,13] or organics [14] detection), and so on. Despite of their superior properties and versatility, CDs’ luminescence properties are sensitive to various factors, which greatly limits their optical applications [15,16,17,18]. Recently, extensive studies have been focused on CDs as potential nanosensors for the detection of free Fe^3+^ ions in water systems because Fe^3+^ ions easily form insoluble Fe(OH)_3_ in the neutral physiological environment and are extremely difficult to be assimilated by the human body. Therefore, understanding CDs‘ luminescence behavior and their use in the analysis of Fe^3+^ ions is of great value in environmental monitoring and clinical research [19,20]. 

Based on early studies on the luminescence properties of CDs, it is generally believed that the particle size, [21,22,23,24] solvent polarity [25,26], and surface state [27,28,29,30,31,32,33,34] are mainly responsible for the optical performance of CDs. However, more and more studies emphasize the functional groups on the surface of CDs as the key factor affecting the optical performance of CDs with certain size and specific synthesis conditions [35,36,37]. Nguyen et al. reported that the intense photoluminescence (PL) in CDs was originated from the abundant surface functional groups on the surface rather than the carbon core [38]. Zhu et al. found that the luminescence center of graphene quantum dots was probably induced by the surface state, which was formed by the hybridization structure of edge groups and the connected partial graphene core [39]. Thus, the effects of specific functional groups on optical properties need further investigation [40,41]. Since it is usually inevitable to introduce oxygen and nitrogen elements during the synthesis of CDs, those elements and their related chemical bonds also have great influences on the luminescence properties of CDs. It is of great importance to find an efficient way to separate each chemical state and describe their possible positions within the band structure in order to clarify the characteristic luminescence features in photoluminescence spectra. 

In this work, we report an efficient route to modulate the concentration and categories of the various chemical states of CDs by selecting specific carbon sources. To control the contents and types of intrinsic C (C-C/C=C) and N (graphitic N, pyridine N, and amine N) elements, different amino acids (changing the content of carbon or nitrogen while keeping the content of other elements unchanged) were used as the carbon source, and acid and alkali were used to treat the surface of CDs. The approximate energy level positions of the intrinsic C, graphitic N, pyridine N, amino, and C=O groups were characterized from series of excitation and emission spectra, and the results were consistent with our theoretical calculations. In addition, we have discovered that the as-prepared CDs can be used to selectively detect Fe^3+^ ions in aqueous solution with high sensitivity, which makes CDs a simple and efficient nanosensor for Fe^3+^ ions. 

## 2. Results and Discussion

### 2.1. Synthesis and Characterization of CDs

**Figure 1:**
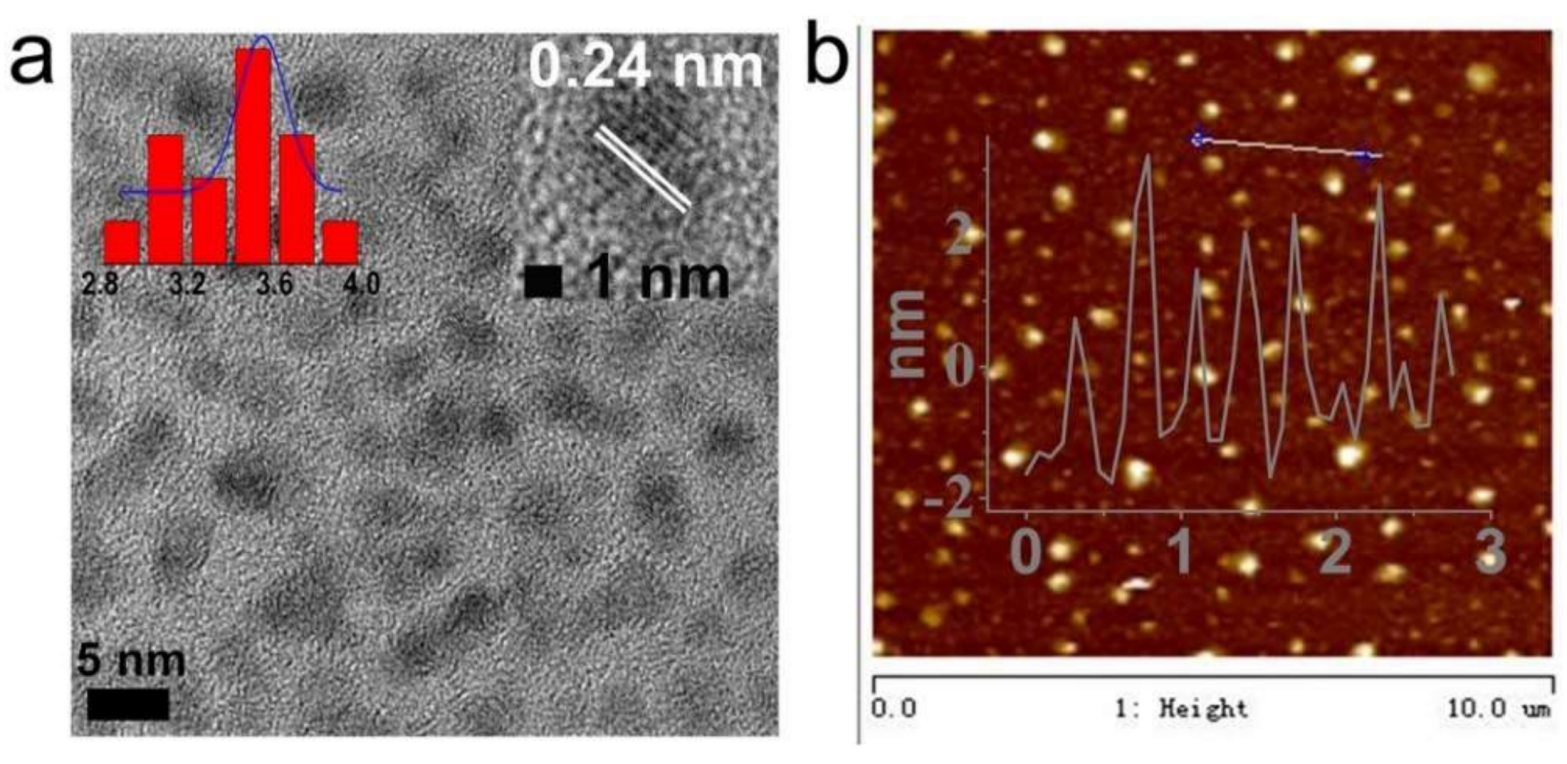
(**a**) Size distribution (inset of a, left) and high-resolution (HR)TEM (inset of a, right) images of CDs-1; (**b**) AFM image and topographic heights of CDs-1.

The CDs with various carbon and nitrogen compositions have been synthesized using different types of raw materals. CDs-1 to 3 were synthesized with increasing carbon contents using glycine (C_2_H_5_NO_2_), alanine (C_3_H_7_NO_2_), and valine (C_5_H_11_NO_2_), respectively. The increase in nitrogen contents from CDs-4 to 6 were obtained by using leucine (C_6_H_13_NO_2_), histidine (C_6_H_9_N_3_O_2_), and arginine (C_6_H_14_N_4_O_2_), respectively. The size and morphology of the synthesized CDs have been characterized by transmission electron microscopy (TEM) and atomic force microscopy (AFM). As illustrated in Figure 1a, the CDs are well dispersed in the solution and have an average size of 3.5 nm. High-resolution TEM images display a lattice fringe of 0.24 nm, which is corresponding to the (1120) planes of graphite [42]. Figure S1 demonstrates a larger view of the distribution for the CDs, from which the particle size is relatively uniform in a wide range. The topographic heights of CDs have been revealed to be between 3 and 4 nm in Figure 1b indicating the CDs are primarily consisted of a few layers of grapheme-like sheets [3].

**Figure 2:**
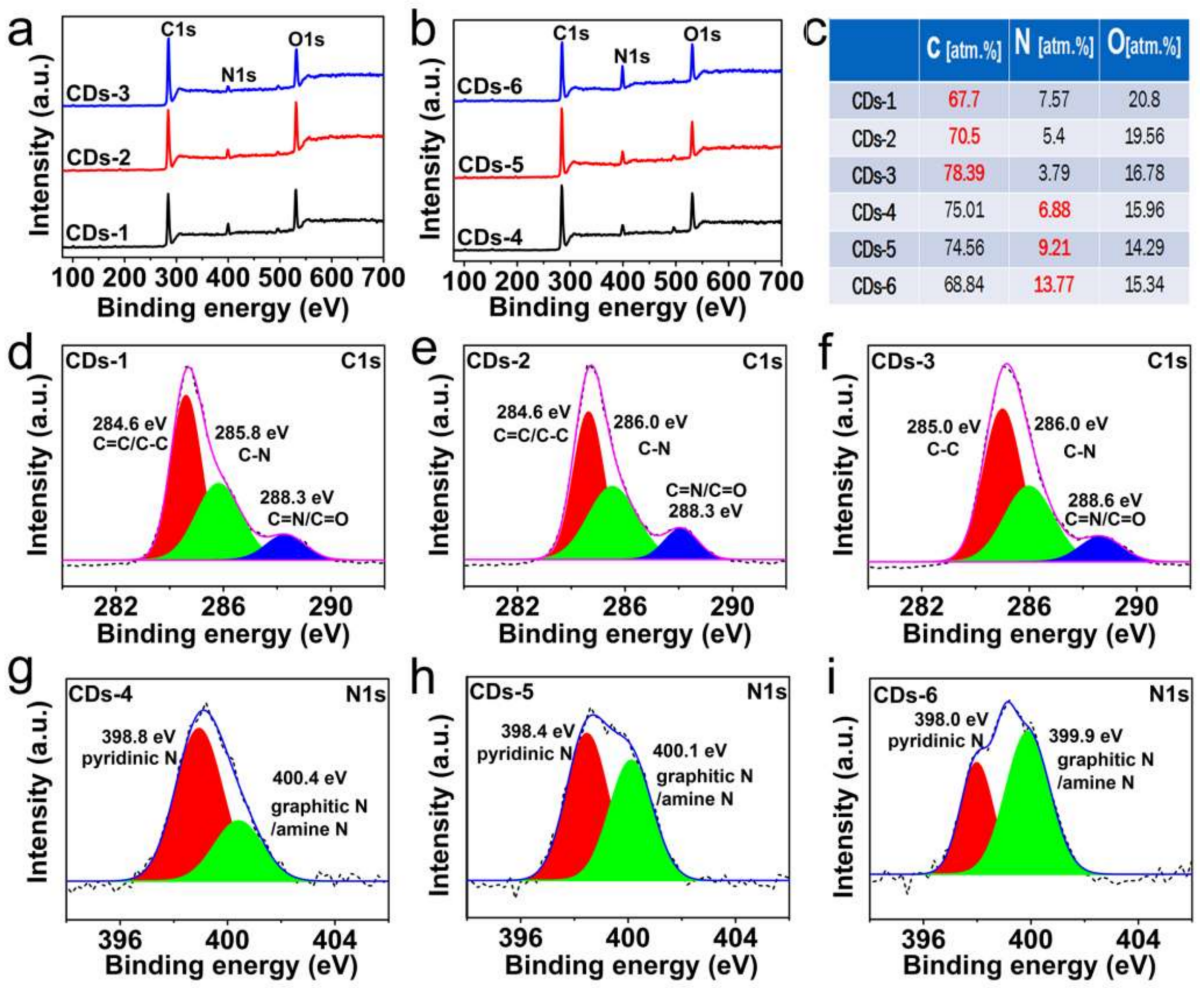
(**a**,**b**) XPS spectra of CDs-1, CDs-2, CDs-3, and CDs-4, CDs-5, CDs-6; (**c**) The element contents for CDs-1 to CDs-6; (**d**–**f**) C1s spectra and fitting results of CDs-1, CDs-2, and CDs-3, respectively; (**g**–**i**) N 1s spectra and fitting results of CDs-4, CDs-5, and CDs-6, respectively.

Fourier transform infrared (FTIR) and X-ray photoelectron spectroscopy (XPS) have been carried out to determine element compositions and related chemical bonding states. FTIR spectra (Figure S2) exhibit characteristic stretching vibrations of O-H and N-H bands centered at 3268 cm^−1^, as evidenced by their vibrational fingerprints at 1518 cm^−1^. C-N stretching vibration is located at 1313 cm^−1^, and the peaks centered at 1728 cm^−1^ and 1456 cm^−1^ originated from C=O and C=N bonds, respectively [43]. As shown in Figure 2a,b, the XPS spectra of CDs exhibit three distinctive peaks centering at 285.0 eV (C 1s), 400.0 eV (N 1s) and 532.0 eV (O 1s), respectively, which implies the existence of N and O elements in the prepared CDs. With the increase of carbon (CDs-1 to 3) or nitrogen contents (CDs-4 to 6) in the precursor, the relative carbon or nitrogen contents in the CDs are increased (Figure 2c). High-resolution XPS spectra of C 1s (Figure 2d−f) reveal three carbon related features located at 284.6, 286.0, and 288.6 eV, corresponding to C=C/C-C, C=N, and C=O bonds, respectively [44]. The high-resolution N 1s spectra of the CDs (Figure 2g–i) show two pronounced peaks at 398.4 and 400.1 eV, which may be caused by pyridine N, graphitic N, or amino N, respectively [26]. With increasing nitrogen contents in the raw materials, the intensity proportion from graphitic and amimo N increases, whereas that of the pyridinic N decreases. In addition, the peak centered at 400.0 eV gradually shifts to the lower binding energy with the increase of nitrogen contents, indicating that the increase of graphite N is faster than amino N. Both the XPS and FTIR spectra suggest the abundancy of amide, carboxyl, pyridine N, and graphite N in prepared CDs. 

**Scheme 1:**
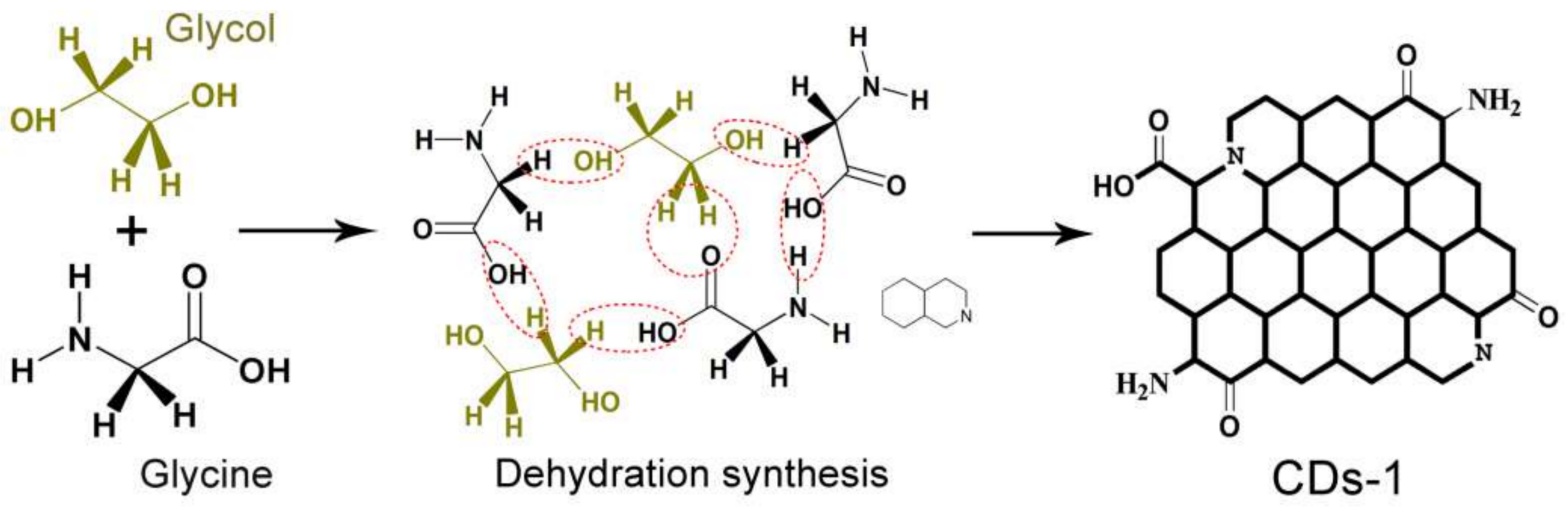
A possible growth mechanism for CDs-1.

Considering elements and functional groups in the CDs, we propose a mechanism to describe the process of synthesis, as illustrated in Scheme 1. Through the intermolecular dehydration among glycol, glycine, and the two amino acids with the aid of ethanol serving as the catalyst. The final product contains carbonyl, carboxyl groups, amino and pyridine N on the surface, and graphitic N in the carbon ring. Meanwhile, the heterogeneity of the surface state distribution also indicates that a fluorescence anisotropy of the prepared CDs.

### 2.2. Effects of Carbon Core on Luminescence Property

**Figure 3:**
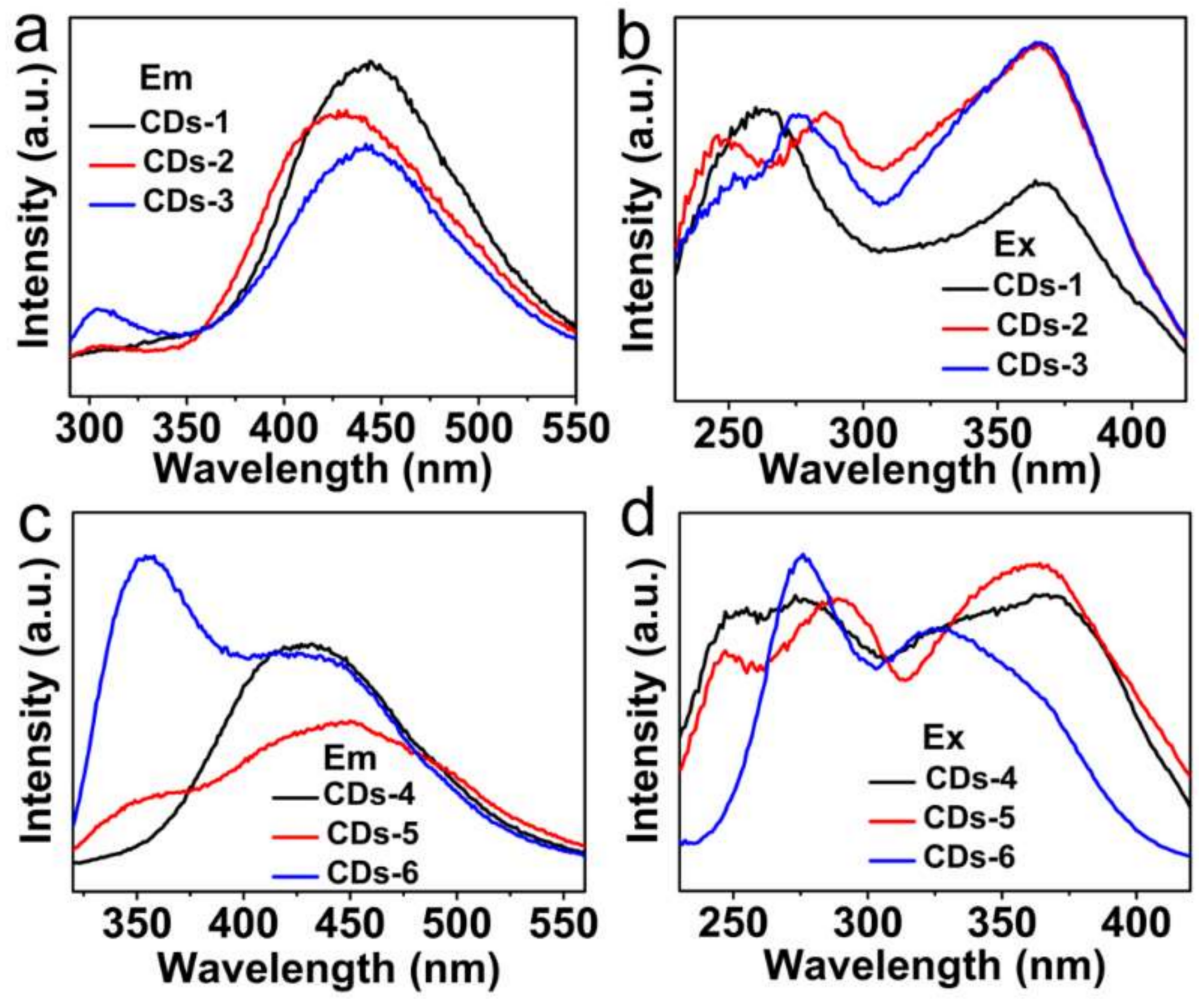
(**a**,**b**) PL emission (Em) spectra (λ_ex_ = 280 nm) and excitation (Ex) spectra (λ_em_ = 445 nm) of CDs-1, CDs-2, and CDs-3; (**c**,**d**) PL emission (Em) spectra (λ_ex_ = 300 nm) and excitation (Ex) spectra (λ_em_ = 445 nm) of CDs-4, CDs-5, and CDs-6.

For CDs, both the carbon core and surface state will affect their luminescence properties. Figure 3 gives the PL emission and excitation (PLE) spectra of CDs-1, CDs-2, CDs-3, CDs-4, CDs-5, and CDs-6. For the emission spectra of CDs-1, CDs-2, and CDs-3 (Figure 3a), there is a broad emission peak centered at 445 nm and a small emission peak centered around 305 nm. The emission peak around 305 nm becomes more obvious with the increase of carbon contents, which means that the emission level here is related to the carbon related groups [3]. For the PLE spectra of CDs-1, CDs-2, and CDs-3 (Figure 3b), there are three excitation peaks located at 250, 275, and 365 nm, respectively. The 275 nm and 365 nm excitation peaks are not sensitive to the carbon contents while the 250 nm excitation peak is related to carbon contents. The PL emission and PLE spectra of CDs-1, CDs-2, and CDs-3 are mainly originated from the C-C and C=C bonds related impurity levels. For the PL emission of CDs-4, CDs-5, and CDs-6, (Figure 3c), there are two emission peaks centered at 355 nm and 445 nm, and the emission peak of 355 nm gradually occupies the leading position from CDs-4 to CDs-6, which may be induced by the increase of graphite N-related impurity levels. In addition, the PLE spectra are also influenced by the nitrogen contents (Figure 3d). Similar to the CDs-1 to CDs-3, there are four excitation peaks centering at 250 nm, 275 nm, 325 nm, and 365 nm for CDs-4 to CDs-6. The relative intensity for excitation peaks of 250 nm and 365 nm is decreased with increasing nitrogen contents and the two excitation peaks are almost disappeared for CDs-6. It can be seen that the excitation peaks located at 275 nm and 325 nm are also related to the N related impurity levels of CDs. According to the above analysis, we can conclude that the emission and excitation spectra are influenced by both carbon and nitrogen contents and related impurity levels in the carbon core and surface, as well as their related energy levels, which will be discussed in Figure 6 in detail.

### 2.3. Effects of Surface States on Luminescence Property

**Figure 4:**
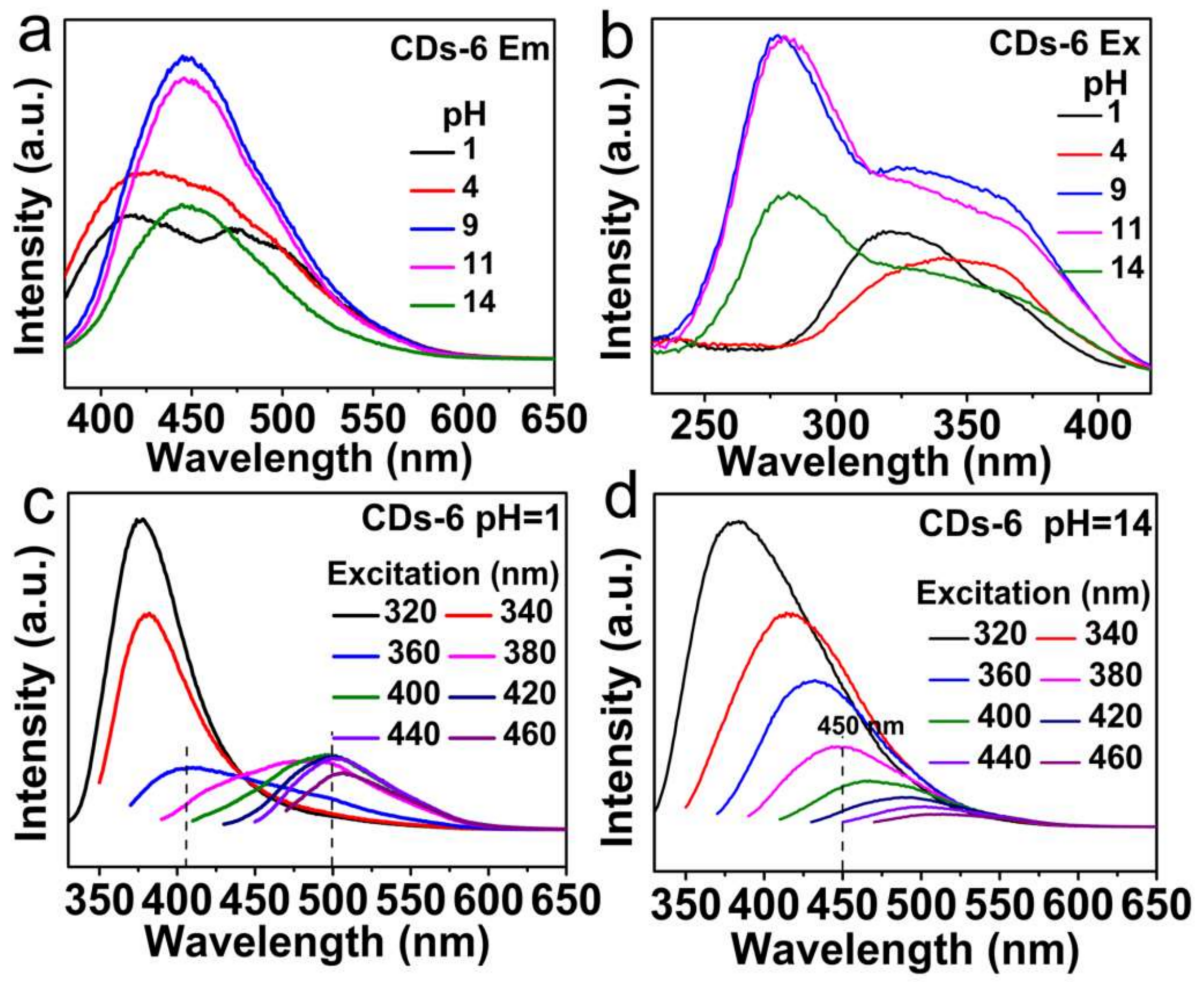
(**a**,**b**) Emission (Em) and excitation (Ex) spectra of CDs-6 by changing pH values (1, 4, 9, 11, 14) with addition of HCl and NaOH; (**c**,**d**) Emission spectra of CDs-6 under different excitation wavelengths in the range of 320‒460 nm for pH = 1 and pH = 14, respectively.

To further investigate the effects of surface state on the PL properties of CDs, the PL and PLE spectra of CDs in acid and alkali solutions with different pH values were investigated. Considering that the surface groups of CDs-6 are relatively abundant due to its maximum nitrogen contents. The amino and carboxyl groups are a pair of special groups. Under acidic conditions, the amino groups are easily protonated, while the carboxyl groups are easily deprotonated in an alkaline environment [45]. Taking CDs-6 as an example, the amino and carboxyl groups on the surface were treated by acid and alkali with pH = 1–14. To eliminate the influence of sodium ions and chloride ions, CDs-6 were treated with strong acid-base (HCl, NaOH, Figure 4) and weak acid-base solutions (CH_3_COOH, NH_3_∙H_2_O, Figure S3), respectively. The consistency of the experimental results ruled out the possible effects of the sodium and chloride ions on the luminescence spectra of CDs. 

From Figure 4a, it can be seen that there is one emission peak centered at 445 nm for pH ≥ 9, and the peak intensity is decreased with the enhancement of solution alkalinity. We found that the treatment of alkaline conditions would not change the position of the emission peak. However, with the increase of the acidity in the solution, the emission peak of 445 nm gradually disappeared, and new emission peaks centered at 410 nm and 500 nm were observed for pH = 1. The increase of acidity mainly affects the amino groups on the surface of CDs, which indicates that the emission peak of 445 nm is mainly related to the surface amino-related impurity levels. With the disappearance of the emission peak at 445 nm, the original broad peaks are separated into two independent emission peaks of 410 nm and 500 nm, which may originate from the surface of pyridine N and C=O-related impurity levels, respectively. The excitation spectra of CDs are also changed as the solution becomes more acidic or more basic, as shown in Figure 4b. There are three excitation peaks centered at 275 nm, 325 nm, and 365 nm for pH ≥ 9 in the excitation spectra, respectively. Consistent with the emission spectra, the excitation peak centered at 275 nm gradually disappears with the increase of the acidity of the solution, disclosing that the excitation of 275 nm and the emission of 445 nm are related to the surface amino groups. Simultaneously, the excitation peaks of the 325 nm and 365 nm are gradually dominated. 

Figure 4c,d shows the emission spectra of CDs-6 under different excitation wavelengths in strong acid and basic solution with pH = 1 and 14, respectively. The emission spectra display different features under various excitation wavelengths. For pH = 1 (Figure 4c), the emission peak is centered around 375 nm for λ_ex_ = 320 and 340 nm (graphite nitrogen related impurity levels dominated), and the emission peak is shifted to 410 nm for λ_ex_ = 360 nm (pyridine N related impurity levels dominated). With increased excitation wavelength, the emission peak position is located around 500 nm (C=O related impurity levels dominated) and the intensity of emission peak near 450 nm is greatly decreased due to the reaction of amino groups with hydrogen ions under acidic conditions. For pH = 14 (Figure 4d), the emission spectra of CDs-6 show strong dependence on excitation wavelength, and emission peak position is red-shifted from 375 to 520 nm accompanying decreased peak intensity, which is induced by intrinsic impurity levels (related to graphitic N) and the impurity levels (related to pyridine N, amino N, and C=O groups) [27,33]. The excitation dependence of CDs suggests that there are many emission levels in the carbon nucleus and the surface. As the 450 nm emission level is destroyed, the longer wavelength emission has certain excitation independence, indicating the existence of energy transfer between the various levels.

### 2.4. Theoretical Calculations

**Figure 5:**
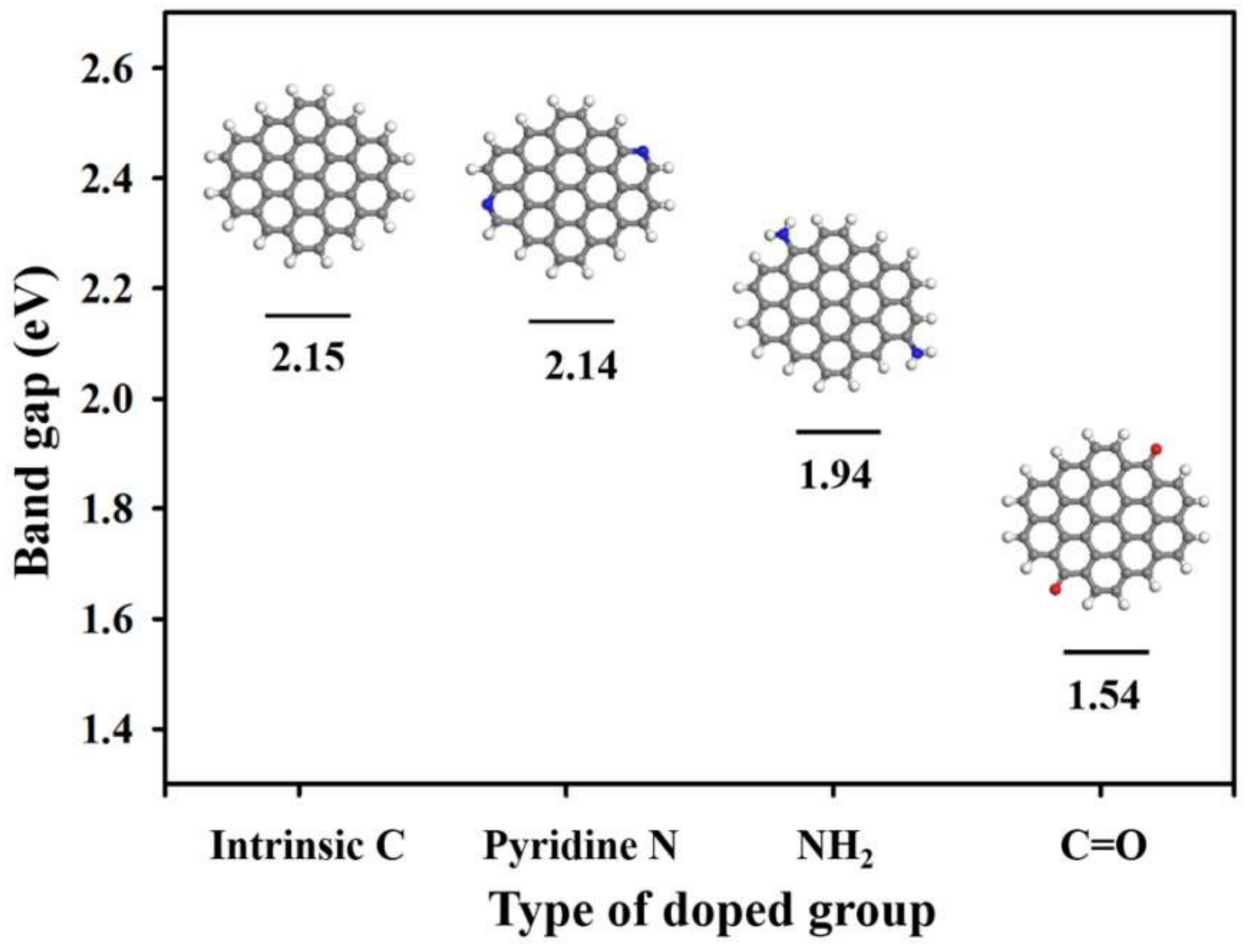
Band gap change of CDs as a function of the doped groups (inset images are optimized structures of CDs) (C: gray; N: blue; O: red; H: white).

To further investigate the luminescence mechanism of CDs, density functional theory calculations were carried out at the HSE06/6-31G* level of theory. A finite carbon nanoflake containing 14 aromatic rings with the edges passivated by hydrogen atoms was used to represent pristine CDs, and its size is similar to that used in the literature [24]. According to our experimental results, the edges of CDs are doped with two pyridinic nitrogen atoms, two amino groups or two carbonyl groups to simulate the carbon core and various surface states. The bond length of ca. 1.42 Å (Figure 5) is in good agreement with the characteristic value of sp2 carbon in graphene. The band gap of our CD model was calculated to be 2.15 eV. Figure 5 shows the evolution of calculated gap energy as a function of dopant types. Clearly, the band gaps are gradually decreased to 2.14, 1.94, and 1.54 eV with doping of pyridine N, amino N, and C=O groups, respectively. It is well known that the band gap is underestimated by the generalized gradient approximation methods, but the relative order should be correct. Therefore, it is expected that the doping can create band gaps and result in a pronounced modification of the electronic structures and optical properties of CDs. It is noteworthy that the much narrower band gaps of surface states (pyridine N, -NH_2_, C=O) than that of the carbon core (intrinsic C) suggest that they are critical for the regulation of the luminescence spectra. 

### 2.5. Energy Band Structure of CDs

**Figure 6:**
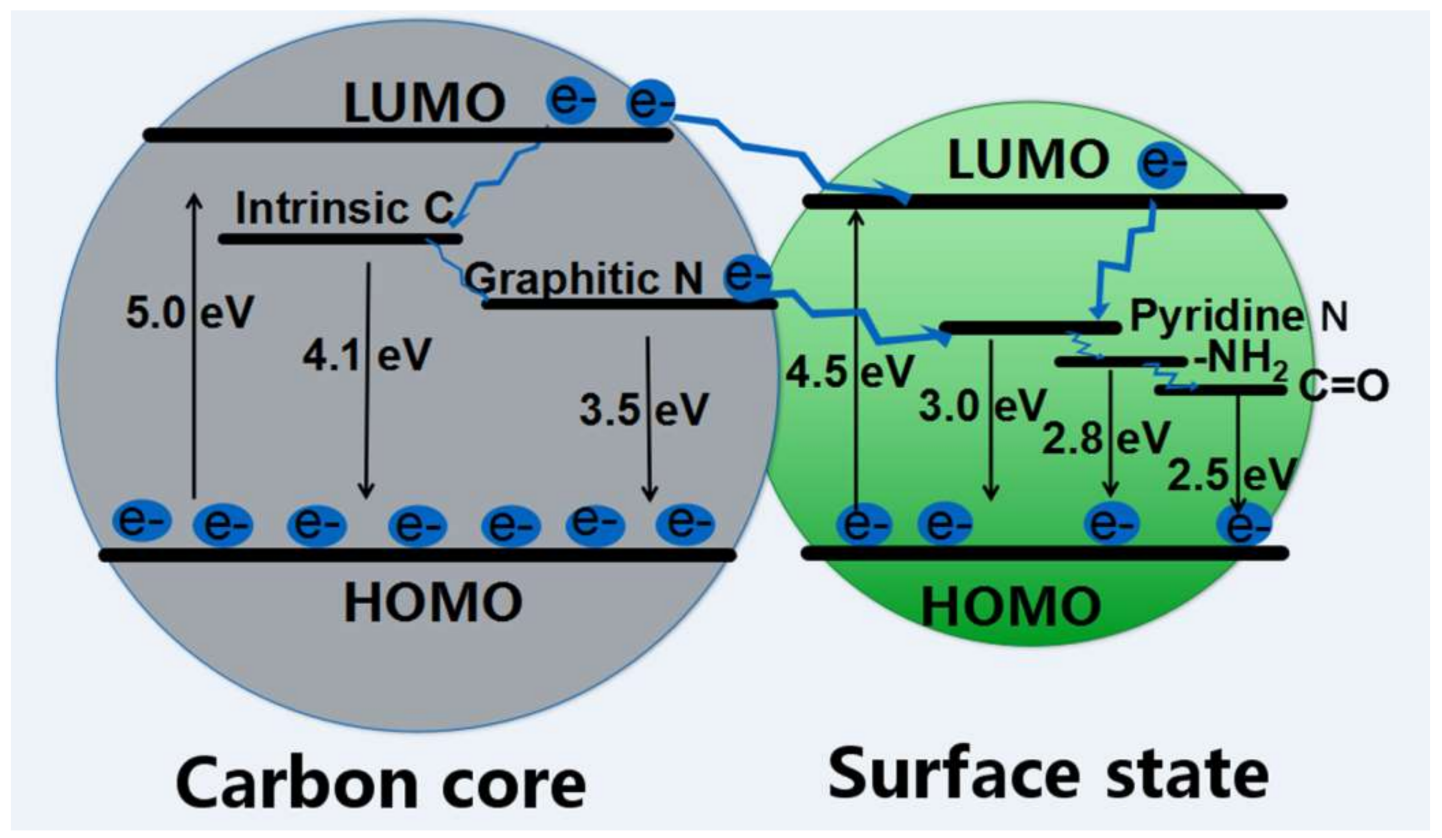
A scheme of energy band structure and possible luminescence process for CDs.

According to the XPS, FTIR, and PL spectra of CDs, as well as the computational results, a scheme of energy band structure of CDs was proposed, as shown in Figure 6. The excitation peak of 255 nm is probably attributed to the transition from the highest occupied molecular orbital (HOMO) to the lowest unoccupied molecular orbital (LUMO) of carbon core (intrinsic C and graphitic N related impurity levels) (Figure 3). Similarly, the excitation peak centered at 275 nm may be attributed to the transition from HOMO to the LUMO of surface state (pyridine N, amino N, and C=O-related impurity levels) (Figure 4). Therefore, the band gap between HOMO and LUMO is about 5.0 eV (λ_ex_ = 250 nm) for the carbon core, whereas that for the surface of CDs is 4.5 eV (λ_ex_ = 275 nm). In addition, the oxygen and nitrogen elements and related chemical bonds will produce impurity levels in the band gap, which leads to the change of excitation and emission spectra of CDs. As shown in Figure 6, there may be an energy transfer process between the carbon core and the surface state. The CDs display five emission bands centered at 305 nm, 355 nm, 410 nm, 445 nm, and 500 nm, which are correlated with the electron transition at intrinsic C (4.1 eV), graphitic N (3.5 eV), pyridine N (3.0 eV), amino N (2.8 eV), and C=O (2.5 eV) related levels, respectively.

### 2.6. Selective Detection of Fe^3+^ Ions with CDs

**Figure 7:**
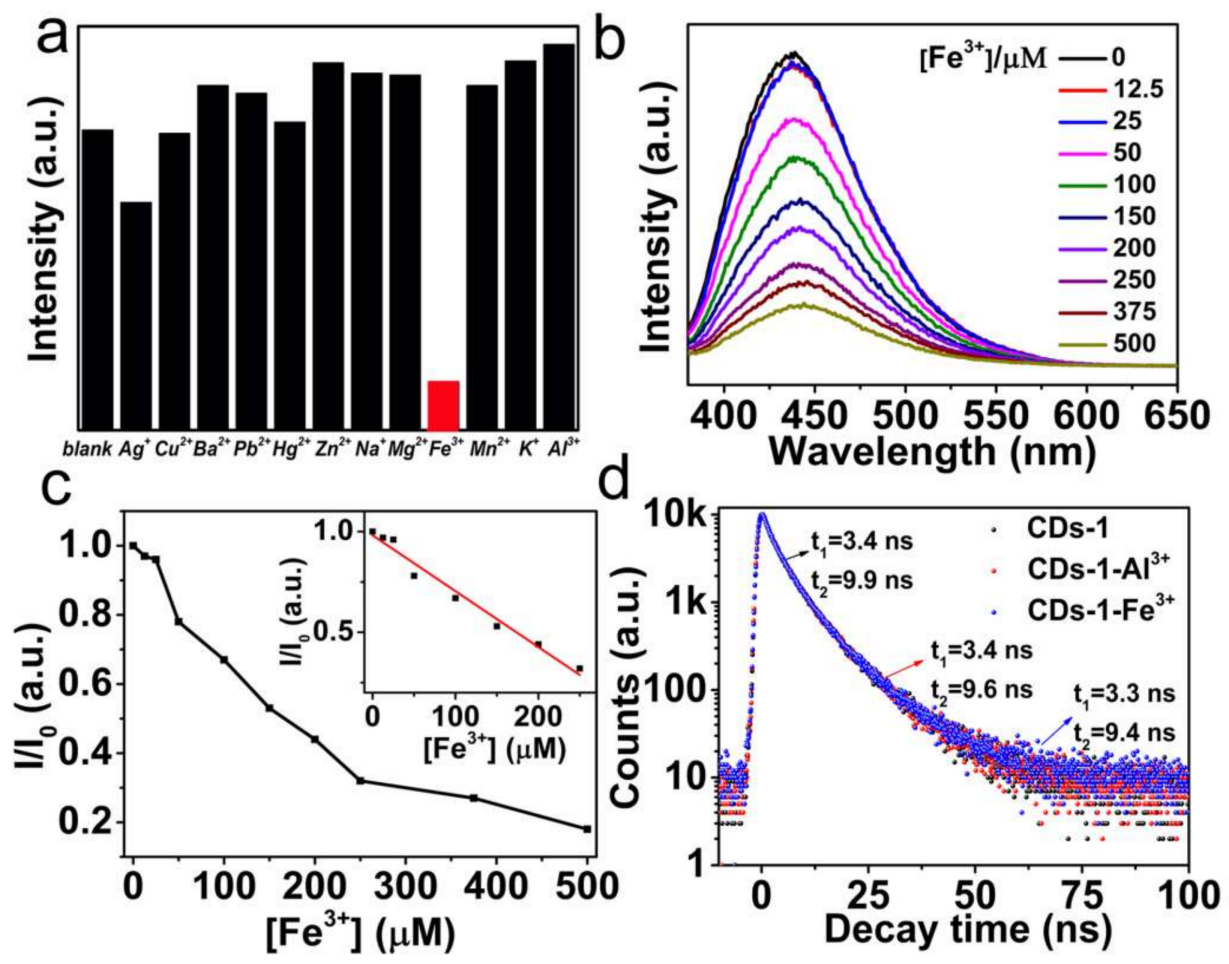
(**a**) Fluorescence intensities of CDs-1 in the presence of different metal ions under 365 nm excitation (the concentration of metal ions and CDs-1 is 500 µM and 1 mg/mL, respectively); (**b**) Emission spectra of CDs-1 with different concentrations of Fe^3+^ ions; (**c**) The plot of I/I_0_ versus the concentration of Fe^3+^ ions, inset: the linear relationship between I/I_0_ and Fe^3+^ ion concentration. (I and I_0_ is the emission peak intensity of CDs-1 under 365 nm excitation with and without Fe^3+^ ions, respectively); (**d**) Fluorescence decay curves and fitting results of CDs-1, CDs-1 with Al^3+^ (CDs-1-Al^3+^), or Fe^3+^ ions (CDs-1-Fe^3+^), respectively.

Figure S4a shows the absorption spectra of the six kinds of CDs (1 mg/mL) and Fe^3+^ ions (500 µM) solutions. It can be seen that CDs-1, CDs-2, CDs-3, and CDs-4 have obvious absorption peaks near 280–330 nm, while the absorption peaks of CDs-5 and CDs-6 become not obvious, which may be induced by the increase of nitrogen contents in CDs-5 and CDs-6. However, it can be seen that the absorption spectrum of Fe^3+^ ions completely covers the absorption peaks of the prepared CDs, suggesting that Fe^3+^ ions may have a fluorescence quenching effect on CDs. Fe^3+^ ions are not normally metabolized in the human body, and excess Fe^3+^ ions can cause cell canceration. Therefore, the detection of Fe^3+^ ions is very important. In order to clarify the singleness fluorescence sensing of CDs for Fe^3+^ ions, the quenching performance of 12 kinds of metal ions (500 μM) was tested with the excitation wavelength of 365 nm. Figure 7a shows the emission peak intensity of CDs-1 with various metal ions, including Ag^+^, Cu^2+^, Ba^2+^, Pb^2+^, Hg^2+^, Zn^2+^, Na^+^, Mg^2+^, Fe^3+^, Mn^2+^, K^+^, and Al^3+^ ions. The results indicate that Fe^3+^ ions have the highest degree of quenching for CDs-1 compared with other metal ions. The other five kinds of CDs (including CDs-2, CDs-3, CDs-4, CDs-5, and CDs-6) also display similar characteristics as CDs-1 (Figure S5). Figure 7b gives the emission spectra for CDs-1 with different concentrations of Fe^3+^ ions. With an increasing concentration of Fe^3+^ ions, the fluorescence intensity of CDs-1 is gradually decreased, and the shape and position of the emission peak are not changed. I/I_0_ has good linearity with a correlation of 0.98176 in the concentration range of 12.5–250 μM (Figure 7c). The above results reveal that CDs-1 can be applied to selective detection of Fe^3+^ ions in the aqueous solution by using the fluorescence sensing method. In addition, we studied the quenching mechanism of CDs-1 by Fe^3+^ ions. From the fluorescence decay curves (Figure 4d), we can find that the existence of Fe^3+^ ions (quenched emission) or Al^3+^ (non-quenched emission) ions do not change the fluorescence lifetimes of CDs. From Figure 3 and Figure S4a, it can be seen that the absorption peak of Fe^3+^ ions overlaps well with the excitation peak of CDs, suggesting that fluorescence quenching may be related to the fluorescence resonance energy transfer or the fluorescence inner filter effect. Since the lifetimes of CDs-1 both in the absence and presence of Fe^3+^ ions remain unchanged, the fluorescence resonance energy transfer is unreasonable [46]. In addition, we found that the absorption intensity of CDs-1 with Fe^3+^ ions was increased with increasing concentration of Fe^3+^ ions (Figure S4b). Therefore, it is speculated that the quenching of CDs-1 by Fe^3+^ ions is induced by the fluorescence inner filter effect. From Figure S6b, it can be seen that the absorption of Fe^3+^ ions completely covers that of CDs (including CDs-*x*, *x* = 1, 2, 3, 4, 5, 6), further confirming the existence of a fluorescence inner filter effect between Fe^3+^ ions and CDs, and all these CDs can be applied for selective sensing of Fe^3+^ ions.

## 3. Conclusions

A series of blue light emitting CDs, 2–4 nm in diameter, have been successfully prepared by a facile microwave method. Through using various amino acids as raw materials, the carbon and nitrogen compositions and related chemical bonds have been carefully controlled in order to modulate the luminescence from diffrent trapping states within the band structure of CDs. Those characteristic luminescence features would be associated with carbon and nitrogen related chemical bonds, as well as the functional groups on the surface of CDs. In addition, the excitation and emission spectra also indicate that acid or base solution could affect the optical properties of CDs. Combined with the theoretical calculation results, we proposed a scheme of the band structure pointing out the possible electron transitions from trapping states between the band gap, which leads to the fine features of luminescence from CDs. Moreover, the prepared CDs have a unique selective Fe^3+^ ion sensing effect among other metal ions due to a fluorescence inner filter effect between Fe^3+^ ions and CDs. 

## 4. Experimental Section

### 4.1. Materials

Glycine, alanine, valine, leucine, lysine, arginine, ethanol, ethylene glycol, and FeCl_3_ were purchased from Tengyin Trading Company of Tianjin City, China.

### 4.2. Synthesis of CDs-1, CDs-2, CDs-3, CDs-4, CDs-5, and CDs-6

The CDs (carbon sources are glycine (CDs-1), alanine (CDs-2), valine (CDs-3), leucine (CDs-4), lysine (CDs-5), and arginine (CDs-6), respectively) were synthesized by a facile, low-cost, and one-step microwave approach. All CDs were prepared in the same way. Taking CDs-1 as an example, glycine (5 mmol) was first dissolved in a mixed solution including deionized water (5 mL), absolute ethyl alcohol (2 mL), and ethylene glycol (10 mL), which was stirred until the solution clarified. The solution was then heated in a microwave oven (560 W, 180 °C) for 2 min. The post-microwave solution was cooled to room temperature and then dialyzed for two days using a dialysis membrance (1000 Da) with deionized water. Finally, a clear yellow-brown aqueous dispersion containing CDs was obtained.

### 4.3. Solution of Fe^3+^ Ions with CDs

First, 10 mL deionized water was used to dissolve FeCl_3_ with different masses to form a series of Fe^3+^ ions solution with different concentrations (12.5–500 μM). Then 1 mL Fe^3+^ ion solution and 3 mL CD solution were mixed to form a uniform solution. Finally, the fluorescence spectra of the mixed solution with different concentrations of Fe^3+^ ions were measured under 365 nm excitation.

### 4.4. Computational Method

Density functional theory calculations were carried out using the screened exchange hybrid density functional of Heyd, Scuseria, and Ernzerhof (HSE06) [47] with the standard 6–31G* basis set for all the atoms as implemented in the Gaussian 09 software package [48].

### 4.5. Characterizations

The morphology of CDs was characterized by high resolution transmission electron microscopy (JEOL 2100, Japan Electronics Corporation, Tokyo, Japan). The thickness and morphology was measured by atomic force microscope (Bruker, Nanoscope V, Bruker company, Karlsruhe, German). X-ray photoelectron spectroscopy (PHI1600EXCA, ULVCA-PHI company, Minnesota, USA), and Fourier transform infrared spectroscopy (Bruker, WQF‒410, Bruker company, Karlsruhe, German) were used to characterize the chemical bonding status of CDs. All fluorescence spectra were obtained by a steady and transient state spectrophotometer (Horiba, FL‒3‒22, Hitachi Corporation, Tokyo, Japan).

## Supplementary Materials

This material is available free of charge via the Internet at http://www.mdpi.com/2079-4991/8/4/233/s1. Details of the TEM image for CDs-1, the FTIR for CDs-*x* (*x* = 1 − 6), the PL spectra of CDs-6 under weak acids and weak basic condition, fluorescence intensities of CDs-*x* for different metal ions, and the UV–VIS absorption spectra of CDs-*x* with the addition of Fe^3+^ ions are available in supporting information.
