# Supplementary material for: Luminescence Mechanism of Carbon Dots by Tailoring Functional Groups for Sensing Fe3+ Ions
Source: Nanomaterials (Basel). 2018 Apr 12;8(4):233. doi: 10.3390/nano8040233 (PMC5923563; doi:10.3390/nano8040233)
Supplement: Supplementary file 1 [file nanomaterials-08-00233-s001.pdf]

Luminescence Mechanism of Carbon Dots by Tailoring Functional Groups for Sensing Fe^{3+} Ions

Jingjing Yu[a], Chang Liu[a],Kang Yuan[a], Zunming Lu[a], Yahui Cheng[b], Lanlan Li[a], Xinghua Zhang[a,*], Peng Jin[a,*],Fanbin Meng[a], Hui Liu[c,*]

[a]*School of Materials Science and Engineering,Hebei University of Technology, Tianjin,China.*

[b]*Department of Electronics and Key Laboratory of Photo-Electronic Thin Film Devices and Technology of Tianjin, Nankai University, Tianjin, China.*

[c]*Tianjin University, Sch Mat Sci&Engn, Inst New Energy Mat, Key Lab Adv Ceram & Machining Technol,MinistEduc, Tianjin, China.*

*Corresponding authors. Tel.: +86 022 60204805, Fax.: +86 022 60202660, Email: zhangxinghua@hebut.edu.cn; china.peng.jin@gmail.com; hui_liu@tju.edu.cn.

Supporting Information

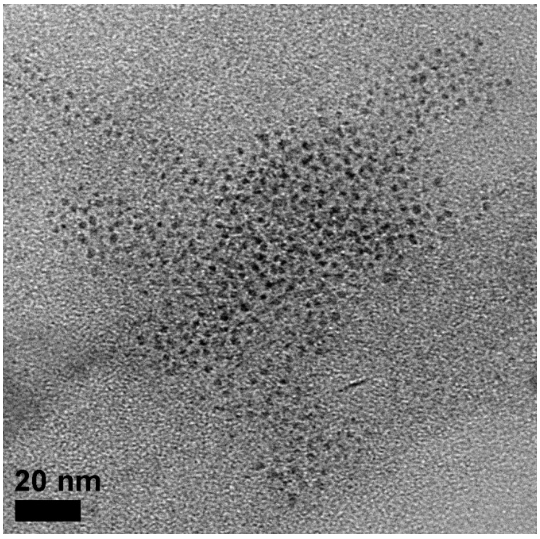

Figure S1. TEM image of CDs-1.

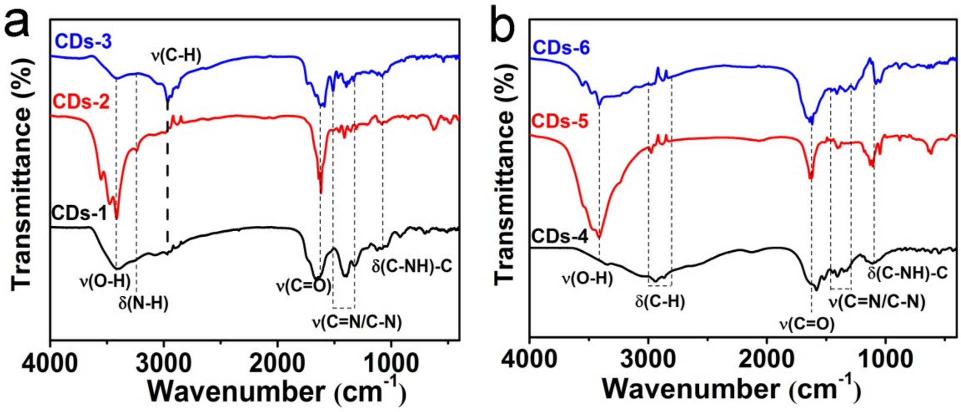

Figure S2. FT-IR spectra of (**a**) CDs-1, CDs-2, and CDs-3; and (**b**) CDs-4, CDs-5, and CDs-6.

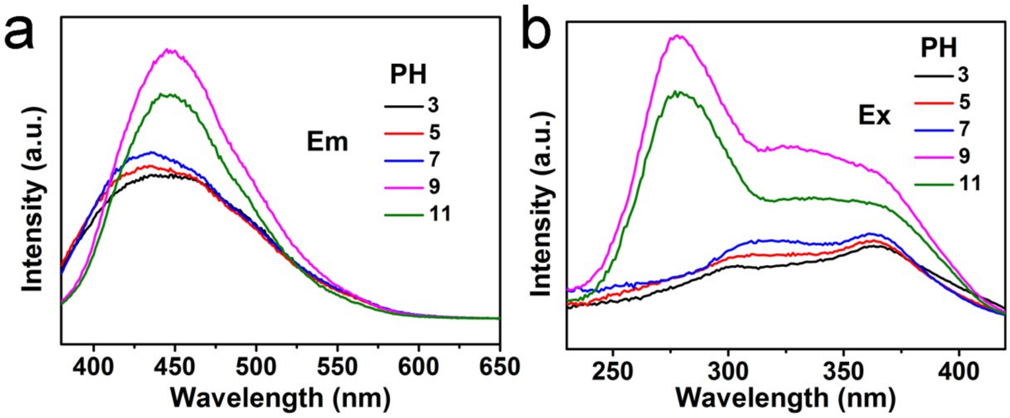

Figure S3. (**a**, **b**) Emission (Em) and excitation (Ex) spectra of CDs-6 by adjustment of different pH values (1, 4, 9, 11, 14) via adding CH_3COOH and $NH_3·H_2O$.

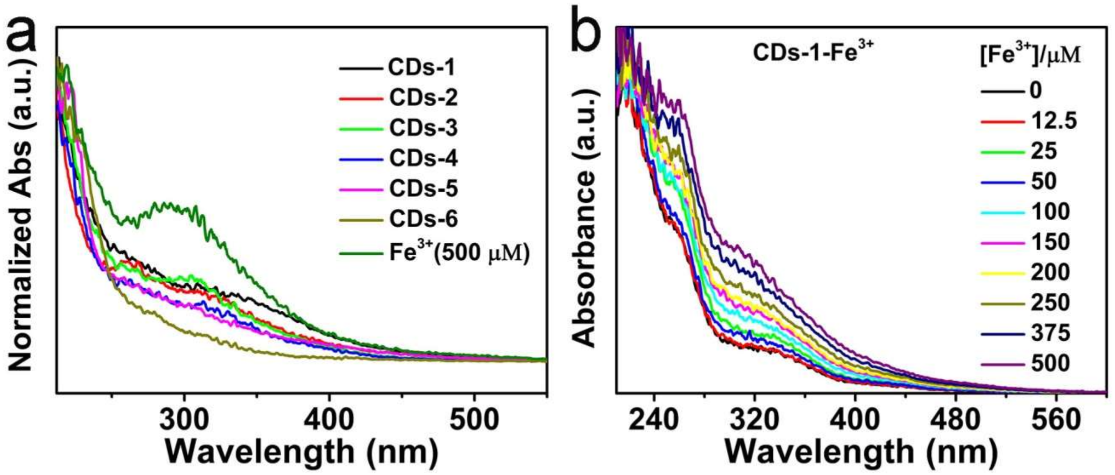

Figure S4. (**a**) Absorption spectra of CDs-1, CDs-2, CDs-3, CDs-4, CDs-5, CDs-6, and Fe^{3+} ion solution. **(b**) Absorption spectra of CDs-1 in the presence of different concentrations of Fe^{3+} ions.

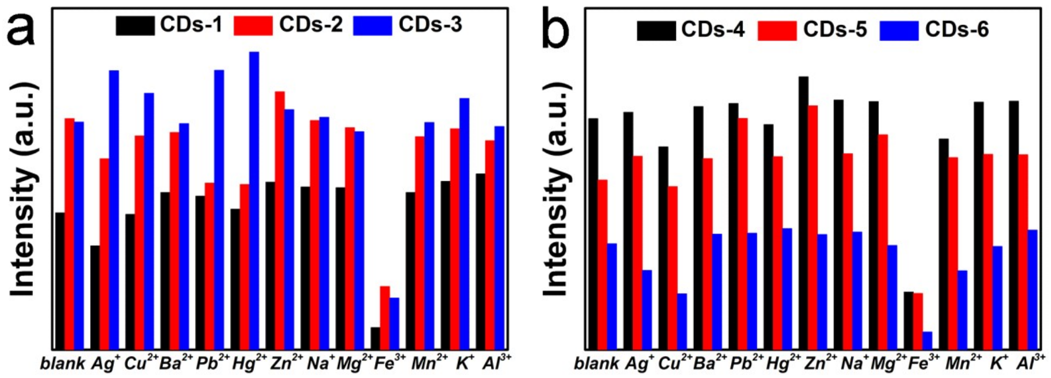

Figure S5. Comparison of fluorescence intensities of CDs under 365 nm excitation (including CDs-*x*, *x* = 1, 2, 3, 4, 5, 6) in the presence of different metal ions (the concentration of metal ions is 500 μM; the concentration of CDs-1 is 1 mg/mL).
